# Single-cell gene profiling of human regulatory T cell subsets in human graft-versus-host disease

**DOI:** 10.1186/1479-5876-9-S2-P28

**Published:** 2011-11-23

**Authors:** Shen Dong, Sylvie Maiella, Aliénor Xhaard, Yuanyu Pang, Christophe Becavin, Arndt Benecke, Gérard Socié, Elisabetta Bianchi, Lars Rogge

**Affiliations:** 1 Immunoregulation Unit, and CNRS URA 1961, Institut Pasteur, Paris, France; 2 Service d'Hématologie Greffe, AP-HP, Hôpital Saint-Louis, Paris, France; 3 Institut des Hautes Études Scientifiques and CNRS USR 3078, Bures sur Yvette, France; 4 Inserm U728, Paris, France

## Introduction

Acute graft-versus-host disease (aGVHD) is the main complication of hematopoietic stem cell transplantation (HSCT) and is an important cause of mortality after HSCT. Regulatory T cells (Treg) therapy has proven highly effective in experimental mouse models of GVHD; however, translation of this approach into humans has been difficult because of the identification of several distinct subsets of human Treg and the observation that an inflammatory environment may cause conversion of human Treg into effector Th17 cells.

## Results

Based on the expression of CD45RA and HLADR, we have identified three different subsets of human FOXP3^+^ Treg in peripheral blood or cord blood, which present suppressive activity *in vitro.* Gene expression profiling combined with global pathway analysis revealed clearly distinct immune signatures for each subset, which were validated by analysis at the single-cell level. Single-cell gene profiling also revealed a striking heterogeneity of gene expression within these Treg subpopulations and that cytokine-expressing Treg did not downregulate FOXP3 and other Treg markers.

We prospectively studied Treg subsets in alloHSCT recipients’ peripheral blood. We found that percentages of FOXP3^+^ cells were not significantly different in aGVHD patients and in the control group. However, a strong alteration of Treg subsets was observed in the aGVHD group compared to the control group, with a pronounced bias towards an activated phenotype, while naïve Treg were almost absent. Gene expression analysis of the three populations at the single-cell level shows a stable expression of Tregs markers.

## Conclusion

As human Tregs constitute a heterogeneous population, the analysis of specific Treg subsets frequencies rather than total pool of CD4^+^FOXP3^+^ Treg frequency may be more refined. We propose that an excess of activated Tregs may serve as biomarker for aGVHD.

